# Mesoporous silica microparticles gated with a bulky azo derivative for the controlled release of dyes/drugs in colon

**DOI:** 10.1098/rsos.180873

**Published:** 2018-08-15

**Authors:** Daniel Ferri, Pablo Gaviña, Margarita Parra, Ana M. Costero, Jamal El Haskouri, Pedro Amorós, Virginia Merino, Adrián H. Teruel, Félix Sancenón, Ramón Martínez-Máñez

**Affiliations:** 1 Instituto Interuniversitario de Investigación de Reconocimiento Molecular y Desarrollo Tecnológico (IDM), Universitat de València-Universitat Politècnica de València, Valencia, Spain; 2 CIBER de Bioingeniería, Biomateriales y Nanomedicina (CIBER-BBN), Madrid, Spain; 3 Departamento de Química Orgánica, Universitat de València, Doctor Moliner 50, Burjassot, 46100 Valencia, Spain; 4 Departamento de Química, Universitat Politècnica de València, Camí de Vera s/n, 46022 Valencia, Spain; 5 Instituto de Ciencia de los Materiales (ICMUV), Universitat de València, Catedrático José Beltrán, 2, Paterna, 46980 Valencia, Spain; 6 Pharmacy and Pharmaceutical Technology and Parasitology, Universitat de València, Burjassot, 46100 Valencia, Spain

**Keywords:** mesoporous silica microparticles, gated materials, controlled drug release, colon targeting, inflammatory bowel disease, budesonide

## Abstract

Mesoporous silica microparticles were prepared, loaded with the dye safranin O (**M-Saf**) or with the drug budesonide (**M-Bud**) and capped by the grafting of a bulky azo derivative. Cargo release from **M-Saf** at different pH values (mimicking those found in the gastrointestinal tract) in the absence or presence of sodium dithionite (a reducing agent mimicking azoreductase enzyme present in the colon) was tested. Negligible safranin O release was observed at pH 6.8 and 4.5, whereas a moderate delivery at pH 1.2 was noted and attributed to the hydrolysis of the urea bond that linked the azo derivative onto the external surface of the inorganic scaffold. Moreover, a marked release was observed when sodium dithionite was present and was ascribed to the rupture of the azo bond in the molecular gate. Budesonide release from **M-Bud** in the presence of sodium dithionite was also assessed by ultraviolet-visible spectroscopy and high performance liquid chromatography measurements. In addition, preliminary *in vivo* experiments with **M-Saf** carried out in mice indicated that the chemical integrity of the microparticles remained unaltered in the stomach and the small intestine, and safranin O seemed to be released in the colon.

## 1. Introduction

Inflammatory bowel disease (IBD) is a group of chronic inflammatory conditions of the colon and small intestine with alternate periods of relapse and remission [1,2]. Typical clinical symptoms of IBD are abdominal pain, diarrhoea, tenesmus and rectal bleeding, caused by ulcerative proctitis or proctosigmoiditis [3]. IBD is so widespread that it is believed that up to 1% of the population can be affected. Ulcerative colitis (UC) and Crohn's disease (CD) are the most common types of IBD [4,5]. UC affects only the colon and the rectum, whereas Crohn's can affect any part of the digestive tract. UC is a disease that causes inflammation and sores (ulcers) in the lining of the large intestine. It usually affects the lower section (sigmoid colon) and the rectum, but it can affect the entire colon.

UC can be treated with a large number of drugs, including 5-ASA derivatives such as sulfasalazine and mesalazine [6,7]. Immunosuppressive medications such as azathioprine and biological agents such as infliximab and adalimumab are also used, although these treatments are less commonly used owing to risk factors. One of the most important medical treatments of UC involves corticosteroids such as prednisone due to their immunosuppressive and short-term healing properties, but because their risks outweigh their benefits, they are not used in long-term treatments [8,9].

Budesonide is considered a second-generation corticosteroid with improved clinical response in patients with distal UC [10,11]. This drug has greater affinity for the intracytoplasmic glucocorticoid receptors than other corticosteroids, which increases its anti-inflammatory power. However, the direct administration of budesonide allows the drug to be absorbed in the proximal intestine and metabolized in the liver, where more than 90% of the dose reaching the blood is transformed into two inactive metabolites: 16α-hydroxyprednisolone and 6β-hydroxybudenoside [12]. These facts preclude its arrival to the affected areas and give rise to adverse effects.

Several oral and rectal formulations such as those based on the use of multi-matrix system technology have been explored to improve pharmacokinetic roles, but new studies directed towards the preparation of more efficient formulations are being continuously published [13].

On the other hand, mesoporous silica materials (MSMs) have found a large number of potential applications as drug delivery systems [14–19]. MSMs show unique properties such as large load capacity, biocompatibility, high thermal stability, homogeneous porosity, inertness and tuneable pore sizes. In addition, they can be prepared in the form of micro- or nanoparticles [20,21]. Moreover, it is possible to incorporate onto the external surface of MSMs functional organic and biological groups which act as molecular gates, able to be opened or closed on command for controlled-release applications. Based on this concept, a number of gated materials capable of delivering their cargo upon application of physical, chemical or biochemical stimuli have been described [22–25]. These controlled-release materials are promising candidates for a great number of biological, pharmaceutical and medical applications [26–29]. For instance, the design of carriers able to release certain drugs on the site of action to minimize secondary effects is a point of reference in the treatment of different diseases.

As mentioned above, the development of new drug carriers for oral IBD treatment to decrease adverse effects of drugs and enhance drug efficacy is a field of interest. In this scenario, formulations for targeting the colon based on pH-responsive polymers, pressure-responsive systems, osmotic-controlled and bio-adhesive materials, time-dependent formulations, prodrugs activated with enzymes or drugs coated with enzyme-sensitive polymers have been reported [30–32]. In particular, the use of enzymes produced by colon microbiota (azoreductase, ß-galactosidase, nitroreductase, etc.) as triggers for drug release targeting is appealing [33]. However, although MSMs capped with different groups have been reported to deliver their cargo by enzyme degradation [34], the use of such systems for the specific delivery of drugs (such as budesonide) in the colon for the treatment of IBD has been barely explored [35,36].

**Scheme 1.**
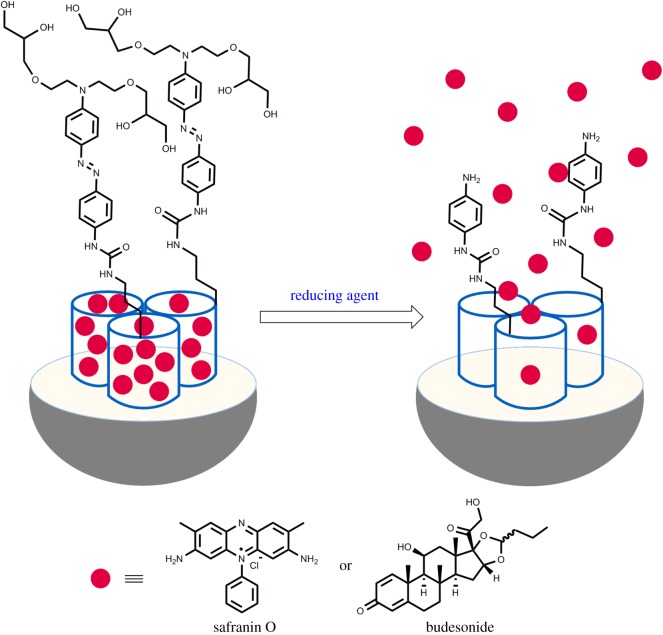
Representation of micrometric silica mesoporous support loaded with safranin O (**M-Saf**) or budesonide (**M-Bud**) and capped with a bulky azo derivative. Cargo is released in the presence of sodium dithionite (a reducing agent).

Following our interest in the use of gated mesoporous silica materials for controlled-release applications, we have recently reported the synthesis of magnetic mesoporous silica microparticles, loaded with a dye and capped with an azo derivative bearing a carbamate linkage, for the release of their cargo in the colon under reducing media [37]. The microparticles showed negligible dye release at neutral pH although a sustained payload release was observed at acidic pH (2.0 and 4.5), ascribable to the hydrolysis of the carbamate bond. Herein we describe the synthesis of simple mesoporous silica microparticles loaded with a dye (safranin O) or with a drug (budesonide), and capped with a bulky azo derivative, for the controlled release of the dye/drug in the colon, triggered by the azoreductases produced by colon microbiota [38]. The azo derivative molecular gate has been attached to the surface of the microparticles through a urea linkage, which is more resistant towards acidic hydrolysis. We found that the presence of the bulky azoderivative on the external surface of **M-Saf** (loaded with safranin O) and **M-Bud** (loaded with budesonide) inhibited cargo delivery, yet both solids were able to deliver their cargo in the presence of a reducing agent (sodium dithionite) (scheme 1) and to some extent at acidic pH (1.2). Moreover, preliminary *in vivo* experiments have been carried out with mice which suggest that the material is able to release the dye in the colon.

## 2. Material and methods

### 2.1. General methods

Nuclear magnetic resonance (NMR), mass spectrometry, powder X-ray diffraction (PXRD), thermo-gravimetric analysis (TGA), transmission electronic microscopy (TEM), N_2_ adsorption–desorption, ultraviolet-visible spectroscopy (UV-vis), fluorescence spectroscopy and high performance liquid chromatography (HPLC) techniques were employed to characterize the synthesized materials. ^1^H and ^13^C NMR spectra were acquired with a Bruker DRX500 spectrometer. High-resolution mass spectra (HRMS) were recorded in the positive ion mode on an AB SCIEX TripleTOF™ 5600 liquid chromatography/mass spectrometry spectrometer. The PXRD measurements were performed with a Bruker D5005 diffractometer by using Cu_K*α*_ radiation. The TGA were carried out on a TG-DTA Pyris Diamond by using an oxidant atmosphere (air 80 ml min^–1^) with a heating programme consisting of heating steps at 5°C per minute from 393 to 1273 K and an isothermal heating step at this temperature for 30 min. The TEM images were obtained with a 100 kV JEM-1010 (JEOL). Grain size distribution was determined by using a Malvern Mastersizer 2000 instrument equipped with a small-volume sample dispersion unit. Water was the dispersion medium used. Each sample was measured in triplicate, accumulating light scatter data for 10 s (in each measurement as well as in the background). To facilitate particle dispersion, samples were sonicated during 2 min at 15 W in successive treatments until stabilization of the particle size distribution. The N_2_ adsorption–desorption were recorded with a Micrometrics ASAP2010 automated sorption analyser. Samples were degassed at 120°C in vacuum overnight. Specific surface areas were calculated from the absorption data in the low-pressure range by using the Brunauer–Ummett–Teller (BET) model. Pore size was determined following the Barrett–Joyner–Halenda (BJH) method. The UV/Vis absorption spectra were recorded in a 1 cm path length quartz cuvette with a Shimadzu UV-2101PC spectrophotometer. Fluorescent spectroscopy studies were carried out with a Varian Cary Eclipse fluorescent spectrophotometer. The HPLC analysis was carried out on a Teknokroma^®^ Brisa LC2 C18 analytical column (5 µm, 150 mm × 0.46 mm) connected to a HPLC (model Flexar Solvent Manager) system (Perkin-Elmer) consisting of a Flexar binary LC pump (Flexar/291N1120202F), diode detector UV (Flexar/292N1111504F) and 20 µl injection loop.

### 2.2. Chemicals

The chemicals tetraethyl orthosilicate (TEOS), *n-*cetyltrimethylammonium bromide (CTAB), sodium hydroxide (NaOH), safranin O, budesonide, dichloromethane, 3-(triethoxysilyl)propyl isocyanate, acetonitrile, hydrochloric acid, 4-nitroaniline, sodium nitrite (NaNO_2_), *N-*phenyldiethanolamine, *p*-toluenesulfonyl chloride, pyridine, tetrahydrofuran (THF), methanol (MeOH) HPLC grade, sodium sulfide nonahydrate (Na_2_S·9H_2_O) were obtained from Aldrich. *n-*hexane and ethyl acetate were purchased from Scharlau. Sodium carbonate (Na_2_CO_3_), magnesium sulfate (MgSO_4_) and ammonium chloride (NH_4_Cl) were purchased from VWR.

### 2.3. Synthesis of **3**

*N-*phenyldiethanolamine (**2**) (4 g, 22.1 mmol) was dissolved in pyridine (40 ml) under argon atmosphere; then *p*-toluenesulfonyl chloride (12.6 g, 66.3 mmol) was added. The reaction mixture was stirred in an ice bath for 5 h. Water was added (150 ml), after which a white solid precipitate appeared. The crude reaction was vacuum-filtered and washed with cold MeOH (15 ml), and dried for 48 h to give pure **3** as a white solid (9.94 g, 92%). ^1^H NMR (300 MHz, CDCl_3_): *δ* 7.71 (d, *J* = 9.0 Hz, 4H), 7.28 (d, *J* = 9.0 Hz, 4H), 7.13 (dd, *J* = 8.8, 7.3 Hz, 2H), 6.70 (t, *J* = 9.0 Hz, 1H), 6.43 (d, *J* = 9.0 Hz, 2H), 4.09 (t, *J* = 6.0 Hz, 4H), 3.55 (t, *J* = 6.0 Hz, 4H), 2.42 (s, 6H) ppm.

### 2.4. Synthesis of **5**

Sodium hydride (198 mg, 5 mmol) was dissolved in dry THF (20 ml) and then the flask was purged several times with argon to remove oxygen and water from the atmosphere of the reaction. DL-1,2-Isopropylideneglycerol (**4**) (560 µl, 4.5 mmol) was gradually added at room temperature. After this addition, the crude reaction was stirred for 1 h at 40°C. Compound **3** (1 g, 2.0 mmol) was dissolved in anhydrous THF (15 ml) and then added dropwise to the crude reaction using a compensated addition funnel. After this addition, the crude reaction was heated at reflux for 24 h. The final brown crude was neutralized with ammonium chloride 10% solution (30 ml) and the organic product was extracted with dichloromethane. Organic layers were dried with anhydrous MgSO_4_ and filtered off, and the solvent was removed under vacuum. Product **5** was purified by silica gel column chromatography and hexane/ethyl acetate (8 : 2 v/v), as a brown oil (560 mg, 68%). ^1^H NMR (300 MHz, CDCl_3_) *δ* 7.20 (dd, *J* = 8.9, 7.2 Hz, 2H), 6.75–6.62 (m, 3H), 4.29–4.21 (m, 2H), 4.03 (dd, *J* = 8.2, 6.4 Hz, 2H), 3.73–3.43 (m, 14H), *δ* 1.41 (s, 6H), 1.36 (s, 6H) ppm.

### 2.5. Synthesis of **7**

4-Nitroaniline (**6**) (170 mg, 1.23 mmol) was dissolved in a mixture of concentrated hydrochloric acid (0.31 ml) and H_2_O (2.5 ml). The resultant solution was cooled in an ice bath to 0°C and stirred, as sodium nitrite (103 mg, 1.5 mmol) in water (0.7 ml) was rapidly added. The crude reaction was stirred at 0°C until a clear solution was formed. This solution was added to a solution of compound **5** (500 mg, 1.23 mmol) in H_2_O (3.6 ml) and concentrated hydrochloric acid (0.2 ml) while maintaining the temperature at below 0°C. The reaction was stirred at 0–5°C for 5 h. The final dark red product was neutralized with a saturated sodium carbonate solution. The red solid was filtered and washed with cold H_2_O. Product **7** was purified by silica gel column chromatography using ethyl acetate/methanol (9 : 1 v/v) as the eluent, to yield a red solid (353 mg, 60%). ^1^H NMR (500 MHz, MeOD-d_4_) *δ* 8.35 (d, *J* = 9.0 Hz, 2H), 7.94 (d, *J* = 9.0 Hz, 2H), 7.87 (d, *J* = 9.0 Hz, 2H), 6.93 (d, *J* = 9.0 Hz, 2H), 3.79–3.71 (m, 10H), 3.60–3.46 (m, 8H) ppm. ^13^C NMR (125 MHz, MeOD-d_4_) *δ* 158.3, 153.6, 148.8, 145.0, 127.2, 125.7, 123.6, 112.9, 73.8, 72.3, 70.1, 64. 5, 52.2 ppm.

### 2.6. Synthesis of **8**

Compound **7** (300 mg, 0.63 mmol) was dissolved in methanol (25 ml), and the solution was heated until reflux. The reducing agent was prepared as follows: sodium sulfide nonahydrate (Na_2_S·9H_2_O) (333 mg, 1.39 mmol) was dissolved in the minimum amount of water and sodium carbonate (107 mg, 1.01 mmol) was added under stirring. This solution was added to the previous one. The resulting solution was stirred at reflux for 2 h. The reaction mixture was poured over ice water. The organic product was extracted with ethyl acetate. Organic layers were dried with anhydrous Na_2_SO_4_ and filtered off, and the solvent was removed under vacuum to give pure **8** as a yellow solid (247 mg, 87%). ^1^H NMR (500 MHz, MeOD-d_4_) *δ* 7.69 (d, *J* = 9.0 Hz, 2H), 7.61 (d, *J* = 9.0 Hz, 2H), 6.85 (d, *J* = 19.0 Hz, 2H), 6.74 (d, *J* = 9.0 Hz, 2H), 3.78–3.74 (m, 2H), 3.71 (s, 8H), 3.60–3.47 (m, 8H). ^13^C NMR (125 MHz, MeOD-d_4_) *δ* 151.9, 151.1, 146.2, 144.9, 125.1, 125.0, 115.5, 112.8, 73.7, 72.3, 70.1, 64.5, 52.1 ppm. HRMS: MH^+^ found: 449.2395, C_22_H_32_HN_4_O_6_ required 449.2403.

### 2.7. Synthesis of **1**

Compound **8** (200 mg, 0.44 mmol) was dissolved in dry THF (20 ml) under argon atmosphere and the solution was heated at 40°C. 3-(Triethoxysilyl)propyl isocyanate (117 µl, 0.47 mmol) was added under stirring. The resultant solution was stirred at reflux for 72 h. The solvent was removed under vacuum to give molecular gate **1** as an orange solid (260 mg). The crude product, which also contained about 30% of the starting amine **8** was used without further purification. ^1^H NMR (500 MHz, DMSO-d_6_) *δ* 8.71 (s, 1H), 7.71–7.67 (m, 4H), 7.52 (d, *J* = 10.0 Hz, 2H), 6.84 (d, *J* = 10.0 Hz, 2H), 6.26 (t, *J* = 5.5 Hz, 1H), 4.64–4.62 (m, 2H), 4,48 (t, *J* *=* 5.8 Hz, 2H), 3.78–3.74 (m, 10H), 3.62–3.57 (m, 10H), 3.47–3.42 (m, 4H), 3.09–3.06 (m, 2H), 1.51–1.48 (m, 2H), 1.15 (t, *J* *=* 7.0 Hz, 9H), 0.59–0.55 (m, 2H). ^13^C NMR (125 MHz, DMSO-d_6_) *δ* 154.87, 146.62, 142.53, 142.28, 131.84, 124.29, 122.78, 117.49, 111.34, 72.57, 70.57, 68.17, 63.04, 57.71, 50.36, 41.78, 23.28, 18.22, 7.26.

### 2.8. Synthesis of mesoporous MCM-41 microparticles

The MCM-41 mesoporous microparticles were synthesized by the following procedure: triethanolamine (TEAH_3_) (25.06 g, 168 mmol) was stirred at room temperature for 5 min. Then, NaOH (12 mmol) in deionized water (2 ml) was added. The reaction mixture was heated at 120°C for 20 min. Next, the solution temperature was adjusted to 70°C. TEOS (10.6 ml, 45 mmol) was then added and was heated to 120°C for 1 h. Then, the solution temperature was adjusted to 118°C and CTAB (4.68 g) was slowly added. Next, the reaction mixture was cooled to 70°C and deionized H_2_O (80 ml) was added with vigorous stirring. After a few minutes, a white suspension resulted. This mixture was aged at room temperature overnight. The resulting powder was collected by filtration and washed with water. Finally, the solid was dried at 70°C (MCM-41 as-synthesized). To prepare the final porous material (MCM-41), the as-synthesized solid was calcined at 550°C using oxidant atmosphere for 5 h in order to remove the template phase.

### 2.9. Synthesis of **M-Saf**

In a typical synthesis, 1 g of MCM-41 mesoporous microparticles and the dye safranin O (500 mg) were suspended in dry acetonitrile (30 ml) under inert atmosphere. The mixture was then stirred for 24 h at room temperature to achieve maximum loading in the MCM-41 scaffolding pores. Next, the solid was filtered off and washed. The solid was resuspendered in dry THF (25 ml). Compound **1** (1 g, 1.44 mmol) was dissolved in anhydrous THF (25 ml) and was added to MCM-41 material loaded with safranin O. Excess safranin O was also added to the mixture to saturate the solution with dye, thus preventing the dye from leaving the inlets of the MCM-41 pores. The mixture was stirred for overnight at room temperature in an argon atmosphere. The solid was isolated by filtration and thoroughly washed with THF, H_2_O until a colourless solution was obtained; then it was dried at 40°C for 12 h to yield **M-Saf** as a dark red-orange solid.

### 2.10. Synthesis of **M-Bud**

In a typical synthesis, 500 mg of MCM-41 and budesonide (500 mg, 1.16 mmol) were suspended in dry acetonitrile (30 ml) under an inert atmosphere. The mixture was then stirred for 24 h at 60°C to achieve maximum loading in the MCM-41 scaffolding pores. Next, compound **1** (500 mg, 0.72 mmol) was added in anhydrous THF (25 ml). The mixture was stirred overnight at room temperature. The solid was isolated by filtration and thoroughly washed with THF, H_2_O until a colourless solution was obtained; then it was dried at 40°C for 12 h to yield **M-Bud** as a yellow solid.

### 2.11. *In vivo* release studies

The studies reported here adhere to the Principles of Laboratory Animal Care and were approved by the institutional ethics committee of the Conselleria de Agricultura, Medio Ambiente, Cambio Climático y Desarrollo Rural (Generalitat Valenciana), according to 2016/VSC/PEA/00158. In a typical experiment, 10 mg of **M-Saf** was suspended in water (200 µl). The mixture was administered to a mouse by oral gavage by using a cannula provided with a rounded tip. The mouse, with free access to water and food, was observed during 4 h, showing a normal behaviour. The mouse was euthanized with an overdose of anaesthesia (dolethal^®^) and then the abdomen was opened and the digestive system was removed. The digestive system was dissected to locate the microparticles through it. The **M-Saf** was observed to pass freely without causing any ulcer or absorption in the other tissues. An appreciable amount of closed material was still present in the stomach; in addition, the microparticles located along the small intestine remained also closed. The material isolated in each section was evaluated using sodium dithionite, and the safranin O delivered was followed by fluorescence spectrometry (figure 5; electronic supplementary material, figure S19). In addition, another mouse was studied during 24 h after oral administration of **M-Saf**. After 24 h the mouse was euthanized in similar conditions and the digestive system was dissected to locate the microparticles through it. After this time, no appreciable amounts of closed material were present in the stomach, intestine or colon, and the corresponding release studies using Na_2_S_2_O_4_ did not show the presence of safranin O in the extracted solutions. In addition, the stool was also evaluated. The stool was homogenized and suspended in water (5 ml) during 24 h at room temperature. The mixture was centrifuged and the supernatant was separated. The solid was suspended again in water (5 ml) and safranin O release was evaluated using sodium dithionite (electronic supplementary material, figure S20).

## 3. Results and discussion

### 3.1. Synthesis and characterization of the gated microparticles

**Scheme 2.**
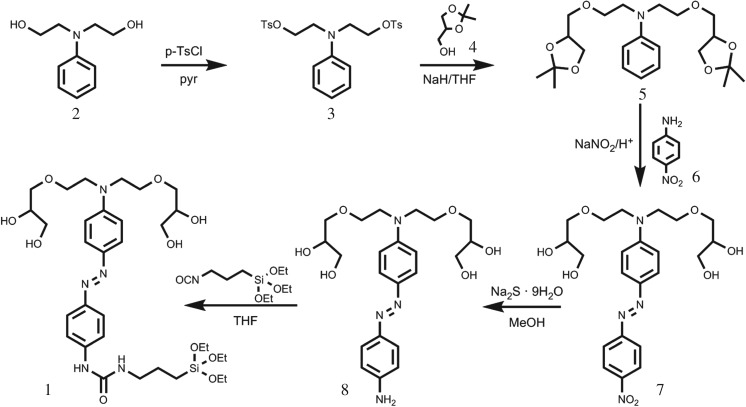
Synthetic route used for the preparation of the capping molecule **1**.

In designing gated supports for a certain application, three issues need to be defined: (i) the capping ensemble used to inhibit cargo delivery, (ii) the trigger that would induce payload release, and (iii) the support containing the cargo [39–41]. In this work, mesoporous silica in the form of microparticles was selected as the scaffold. We selected a standard MCM-41 material instead of other related samples such as uniform mesoporous silica spheres, owing to the larger size of the MCM-41 particles/grains (in the range of several micrometres). The use of microparticles is important for colon targeting to ensure the arrival of the gated material to the area to be treated, avoiding the passage of the material to the bloodstream through the intestine [42]. The selected material contains uniform mesopores in the 2–3 nm range that allow cargo loading; however, the presence of silanol groups on the surface allows anchoring of the capping system through the well-known chemistry of alkoxysilanes [43]. As the capping ensemble a bulky azo-derivative was designed (see molecule **1** in scheme 2), which is expected to be opened by azoreductases produced by the colon microbiota.

The synthesis of **1** is shown in scheme 2. In addition to the cleavable azo group, **1** was designed to contain appended hydroxyl and ether moieties that were expected to be bulk enough to inhibit cargo delivery and, at the same time, to allow water solubility of the residue obtained after the enzymatic rupture of the azo group. Compound **1** also contains a trialcoxysilyl group connected to the azo moiety through a urea spacer, which is resistant to enzymatic hydrolysis [44]. The synthetic sequence followed for the preparation of **1** started with di-tosylation of N-phenyldiethanolamine (**2**), followed by reaction of the di-tosylated derivative **3** with the sodium salt of DL-1,2-isopropylideneglycerol (**4**). These reactions yielded the aniline derivative **5**, which was further coupled with 4-nitroaniline (**6**) by employing sodium nitrite to obtain the azonitro derivative **7**. Reduction of the nitro group using sodium sulfide gave the corresponding amino compound **8**. Finally, reaction of **8** with (3-isocyanatopropyl) triethoxysilane yielded **1**.

Microparticulated mesoporous silica was prepared following well-known procedures by using TEOS, which acts as an inorganic precursor, and CTAB, which serves as a structure-directing agent [45]. The subsequent removal of the surfactant by calcination in air at high temperature yielded the starting mesoporous inorganic support (MCM-41 calcined).

Two final materials, loaded with safranin O (**M-Saf**) and budesonide (**M-Bud**), were prepared using a two-step protocol. In the first step, the pores of the calcined mesoporous scaffold were loaded with safranin O or budesonide by simply stirring a suspension of the microparticles in a solution of the cargo in dry acetonitrile at room temperature (in the case of safranin O) or at 50**°**C (for budesonide) in an argon atmosphere for 24 h. In the second step, the external surface of the microparticles was functionalized by reaction of the trialkoxysilane group in **1** with silanol moieties on the silica surface. This procedure yielded the final loaded gated materials (i.e. **M-Saf** and **M-Bud**) as red-pink and yellow solids, respectively.

**Figure 1.**
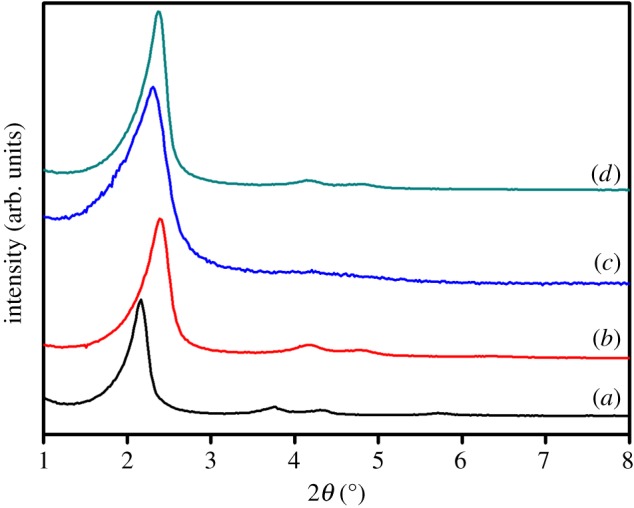
The X-ray diffraction (PXRD) patterns of (*a*) MCM-41 as-synthesized, (*b*) MCM-41 calcined, (*c*) **M-Saf** and (*d*) **M-Bud**.

The starting mesoporous silica scaffold and the final microparticles were characterized by following standard procedures (see the electronic supplementary material for details). Figure 1 shows the PXRD patterns of the microparticulated mesoporous silica as-synthesized and calcined, and the solids **M-Saf** and **M-Bud**. Mesoporous silica microparticles as-synthesized (curve a) displayed four typical low-angle reflections of a hexagonal-ordered matrix indexed at (100), (110), (200) and (210) Bragg peaks. In curve b (calcined), a significant shift of the (100) peak in the PXRD and a broadening of the (100) and (200) peaks were observed. These changes are in agreement with the condensation of silanols in the calcination step. Curves (c) and (d) show the PXRD pattern of solids **M-Saf** and **M-Bud**. For these materials, reflections (110) and (200) were partially lost owing to a reduction in contrast related to the functionalization process and to the filling of mesoporous systems with safranin O (**M-Saf**) or drug budesonide (**M-Bud**). However, the intensity of the (100) peak in these patterns strongly indicates that the mesoporous structure was preserved after the loading process and the additional functionalization with azo derivative **1**.

**Figure 2.**
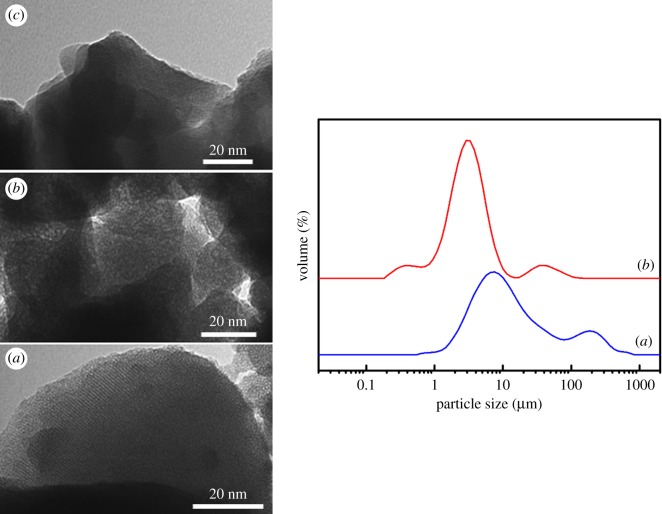
TEM images: (*a*) calcined MCM-41, (*b*) **M-Saf** and (*c*) **M-Bud**. Right: laser diffraction particle size distribution of (*a*) **M-Bud** and (*b*) **M-Saf** materials.

The presence of a mesoporous structure on the starting mesoporous material, **M-Saf** and **M-Bud** was also confirmed by TEM (figure 2, left), where alternate black and white strips or spots (owing to the organized mesopores) can be clearly observed. A lower-order degree is detected for **M-Saf** and **M-Bud** when compared with the starting silica, according to PXRD data. In all cases, the particles have irregular shapes, with a certain aggregation degree leading to grains of micrometric scale. Averaged grain sizes of about 3.2 and 7.2 μm have been determined through laser diffraction (figure 2, right) for samples **M-Saf** and **M-Bud**, respectively.

The N_2_ adsorption–desorption isotherms for calcined mesoporous microparticles and the gated solids **M-Saf** and **M-Bud** were registered and are shown in the electronic supplementary material, figure S9. Curve (a) corresponds to MCM-41 and showed the typical curve for these materials, with an adsorption step at a *P*/*P*_0_ value between 0.1 and 0.3. This corresponds to a type IV isotherm, which is typical of mesoporous materials. This first step is ascribed to nitrogen condensation in the mesopore inlets. The pore diameter (2.56 nm) and pore volume (0.99 cm^3^ g**^−^**^1^) were calculated using the BJH model on the adsorption curve of the isotherm [46]. The absence of a hysteresis loop indicated the cylindrical uniformity of mesopores. The total specific area was 1193.4 m^2^ g**^−^**^1^, calculated using the BET model [47]. In the solids **M-Saf** and **M-Bud**, the N_2_ adsorption–desorption isotherms were characteristic of mesoporous systems with partially filled mesopores (electronic supplementary material, figure S9, curves b and c). A lower N_2_ adsorbed volume and surface area were determined when compared with the initial mesoporous material (electronic supplementary material, figure S9, curve a). This reduction in the BET surface can be explained by the presence of cargo in pores of **M-Saf** and **M-Bud** and the functionalization of the external surface with the azo derivative 1. Table 1 shows a summary of the BET-specific surface values, pore volume and pore sizes calculated from the N_2_ adsorption–desorption isotherms for all the prepared solids. After loading and functionalization, a slight reduction in the accessible mesopore size is observed. The total organic content for **M-Saf** and **M-Bud** solids was determined by thermo-gravimetric analysis and the amount of cargo was determined by elemental analysis and UV-vis spectroscopy (**M-Saf**) (table 2).

**Table 1.** BET-specific surface values, pore volumes and pore sizes calculated from N_2_ adsorption–desorption isotherms for selected materials.

	*S*_BET_ (m^2^ g^−1^)	BJH pore^a,b^ (nm)	total pore volume^a^ (cm^3^ g^−1^)
MCM-41	1193.4	2.56	0.99
**M-Saf**	820.4	2.47	0.52
**M-Bud**	761.2	2.53	0.54

^a^Total pore volume according to the BJH model.

^b^Pore size estimated by using the BJH model applied on the adsorption branch of the isotherm, for *P*/*P*_0_ < 0.6, which can be associated to the surfactant-generated mesopores.

**Table 2.** Total organic matter and amount of cargo (in µg mg^−1^ of solid) for **M-Saf** and **M-Bud** microparticles.

	organic content (µg mg^−1^ material)	cargo (µg mg^−1^ material)
**M-Saf**	260	65
**M-Bud**	260	95

### 3.2. Cargo release studies

Solids **M-Saf** and **M-Bud** contain safranin O and budesonide, respectively, and are functionalized on the outer surface with **1**. The presence of this bulky molecule on the external surface of the solids was expected to inhibit the release of the cargo entrapped inside the pores. Cargo release from **M-Saf** was tested at different pH, simulating the values of gastric juices (1.2), the transition from stomach to intestines (4.5) and that found in the intestine (6.8). In addition, the response of **M-Saf** was also tested in the presence of a reducing agent (sodium dithionite, which mimics azoreductase enzymes present in the colon environment) and of the enzyme nitrate reductase (which is present in the intestinal microflora).

**Figure 3.**
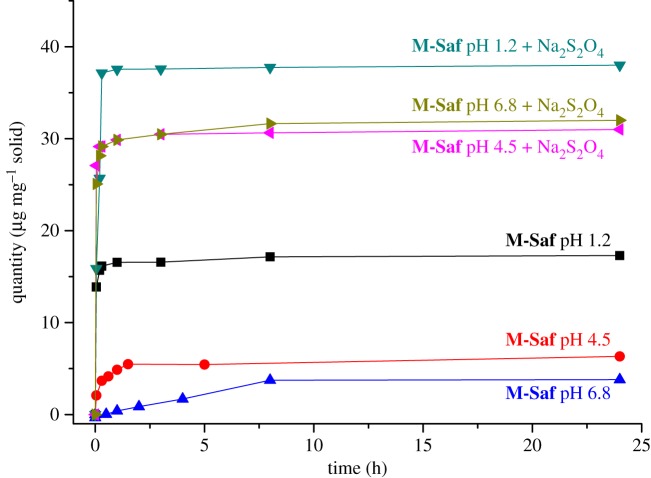
Release profiles of safranin O from **M-Saf** in water at different pH values, in the absence or presence of sodium dithionite.

Dye delivery from **M-Saf** was followed by UV-vis spectroscopy monitoring the safranin O band centred at 520 nm (see the electronic supplementary material). In a typical experiment, **M-Saf** (5 mg) was suspended in water (17 ml) at selected pH (6.8, 4.5 and 1.2) in the absence or in the presence of excess sodium dithionite (3 mg). Aliquots were taken at scheduled times and filtered off in order to eliminate the suspended microparticles, and the absorbances of the resulting solutions were measured to determine the amount of safranin O released from **M-Saf**. The obtained release profiles are shown in figure 3. In the absence of a reducing agent, and at neutral (6.8) or moderate acidic (4.5) pH, negligible safranin O release from **M-Saf** microparticles was observed (maximum delivery of 3.7 and 6.3 µg mg^−1^ solid at pH 6.8 and 4.5, respectively, after 24 h). However, when the pH was set at 1.2, a moderate safranin O release was found (17 µg mg^−1^ solid after 24 h). This delivery is ascribed to a partial hydrolysis of the urea bonds in azo derivative **1** in the highly acidic environment in which the release was carried out. On the other hand, an increased and marked delivery of safranin O from **M-Saf** microparticles at the three tested pH values in the presence of sodium dithionite was observed (figure 3). Nearly the same amount of safranin O at pH 6.8 and 4.5 was observed in the presence of the reducing agent (about 32 µg mg^−1^ solid after 24 h). The higher dye release was observed at pH 1.2 in the presence of sodium dithionite (38 µg mg^−1^ solid, which corresponds to about 60% of dye release, after 24 h). The observed delivery is ascribed to the reductive cleavage of the azo bond in **1** by dithionite, resulting in the opening of the pores and safranin O release to the medium [37]. This cleavage was also confirmed by ^1^H-NMR experiments (see the electronic supplementary material, figure S11). Finally, cargo release studies from **M-Saf** microparticles at neutral pH (7.5) in the presence of nitrate reductase showed negligible safranin O release (data not shown).

**Figure 4.**
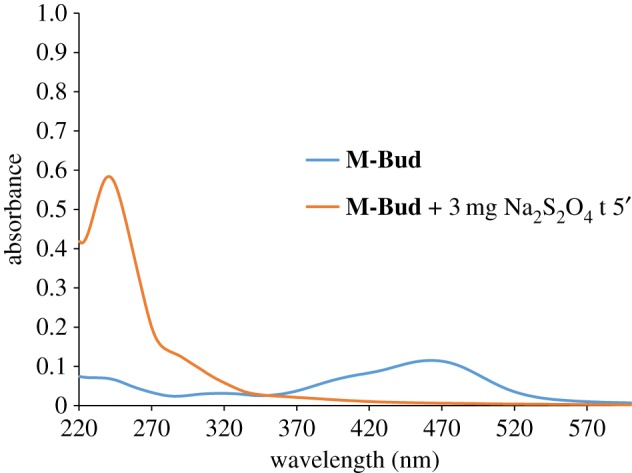
UV-vis spectrum of aqueous suspensions of **M-Bud** in the absence (blue) and in the presence of sodium dithionite (red) after 4 h.

Cargo release from **M-Bud** at neutral pH (not buffered) and in the absence or presence of sodium dithionite was also tested. Budesonide release from **M-Bud** microparticles was followed by UV-vis by monitoring the drug absorption band centred at 241 nm [48]. Aqueous suspensions of **M-Bud** showed a marked absorption band at 463 nm that was ascribed to the azo derivative gate grafted onto the outer surface of the microparticles (figure 4). The intensity of this visible band remained unchanged with time, indicating tight pore closure and negligible budesonide release. However, in the presence of sodium dithionite (1 mM), the absorbance at 463 nm disappeared with time and another band corresponding to budesonide at 241 nm appeared (see again figure 4). The maximum intensity of the 241 nm band was reached 4 h after the addition of the reducing agent.

To determine the amount of budesonide released from **M-Bud**, HPLC studies were carried out. In a typical experiment, **M-Bud** (5 mg) was suspended in water at neutral pH (20 ml) and then an excess of sodium dithionite (3 mg) was added. After stirring at room temperature, aliquots were separated at fixed times, centrifuged to remove the solid and analysed by HPLC [49]. These measurements showed a release of 21.1 µg of budesonide mg^−1^ solid after 5 min with a maximum drug delivery of 31.2 µg mg^−1^ solid after 4 h (see the electronic supplementary material).

### 3.3. *In vivo* release studies

**Figure 5.**
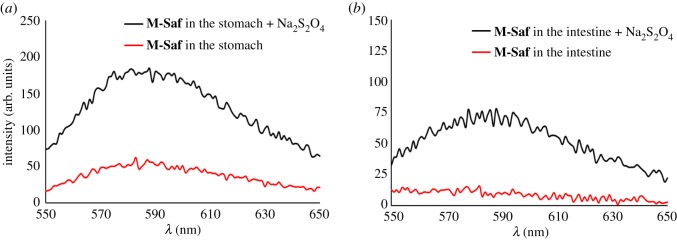
Safranin O emission band (excitation at 520 nm) from aqueous suspensions (1.5 ml) of **M-Saf** nanoparticles isolated from (*a*) stomach and (*b*) intestine, after 4 h of oral administration in the absence and in the presence of sodium dithionite.

Preliminary *in vivo* studies with solid **M-Saf** were carried out with mice in order to evaluate the *in vivo* safranin O release along the entire gastrointestinal tract (GIT). In a typical experiment, **M-Saf** microparticles (10 mg) were suspended in water (200 µl) and then administered to a mouse by oral gavage. After 4 h of **M-Saf** administration, the mouse was euthanized and the digestive system was extracted and dissected in order to ascertain the presence of microparticles along it. As a first conclusion, **M-Saf** microparticles passed through the GIT without causing any ulcer. On the other hand, appreciable amounts of microparticles were present in the stomach and in the small intestine. Then, the microparticles isolated from the different sections of the GIT were suspended in an aqueous solution of sodium dithionite in order to test the preservation of the capping azo derivative **1**. In this regard, when the microparticles isolated from the stomach and small intestine were suspended in water, a negligible emission of safranin O at 550 nm (when excited at 520 nm) was observed (figure 5). On the other hand, when the isolated solids were suspended in water containing the reducing agent, a remarkable emission at 550 nm was found (figure 5). The obtained results suggested that capping azo derivative **1**, attached onto the external surface of **M-Saf** microparticles, retains its chemical integrity on passing through the GIT during, at least, 4 h. The same experiment was repeated but this time the location and integrity of the microparticles were tested after 24 h of **M-Saf** oral administration. After 24 h of the treatment, the mouse was euthanized and the digestive system was studied as before. In this case, unappreciable amounts of microparticles in the stomach and small intestine were observed. In addition, the microparticles found in faeces did not contain the dye (see the electronic supplementary material), which means that it had been presumably released in the last part of the GIT.

## 4. Conclusion

In conclusion, capped silica microparticles loaded with the dye safranin O (**M–Saf**) or the drug budesonide (**M–Bud**) have been prepared and characterized. The capping unit in both materials is a bulky azo derivative which could be broken under reducing conditions (such as those presented in the colon mucosa). Delivery studies at different pH values were undertaken with **M-Saf** using UV–vis spectroscopy. At neutral (6.8) or moderately acidic (4.5) pH, **M-Saf** showed very low safranin-O release. At acidic pH (1.2), a moderate dye release was observed owing to the hydrolysis of the urea bonds that linked the bulky azo derivative onto the external surface of the loaded support. However, in the presence of sodium dithionite (as a mimic of azoreductase enzyme), a marked safranin O release was observed even at neutral pH. This delivery is ascribed to the reductive cleavage of the azo bond, leading to the opening of the pores and subsequent dye delivery. The encapsulation and controlled release of the drug budesonide, used in the treatment of UC, has also been demonstrated with solid **M-Bud** in the presence of sodium dithionite by UV and HPLC measurements. Finally, some preliminary *in vivo* experiments carried out with mice seem to indicate that **M-Saf** retains its chemical integrity in the stomach and the small intestine and it is able to release the dye in the last part of GIT mucosa. We think that these gated microparticles are promising materials for budesonide delivery in the oral treatment of UC.

## Supplementary Material

Supplementary Material

## Data accessibility

The datasets supporting this article have been uploaded as part of the electronic supplementary material.
