# Supplementary material for: Mesoporous silica microparticles gated with a bulky azo derivative for the controlled release of dyes/drugs in colon
Source: R Soc Open Sci. 2018 Aug 15;5(8):180873. doi: 10.1098/rsos.180873 (PMC6124098; doi:10.1098/rsos.180873)
Supplement: Supplementary Material [file rsos180873supp1.pdf]

Electronic Supplementary Material

Mesoporous silica microparticles gated with a bulky azo derivative for the controlled release of dyes/drugs in colon

Daniel Ferri, Pablo Gaviña,* Margarita Parra, Ana M. Costero, Jamal El Haskouri, Pedro Amorós, Virginia Merino, Adrián H. Teruel, Félix Sancenón and Ramón Martínez-Máñez*

Contents:

- ✓ ^{1}H and ^{13}C NMR spectra of compounds
- ✓ Laser diffraction
- ✓ N_2 adsorption-desorption isotherms
- ✓ IR spectra
- ✓ ^{1}H-NMR spectrum of the reductive cleavage of compound **8**
- ✓ Thermogravimetric analysis and elemental analysis
- ✓ HPLC conditions and procedures
- ✓ UV-Vis spectra of dye and drug release studies
- ✓ *in vivo* release studies
- ✓ References

^{1}H and ^{13}C NMR spectra of compounds.

Compound 3.

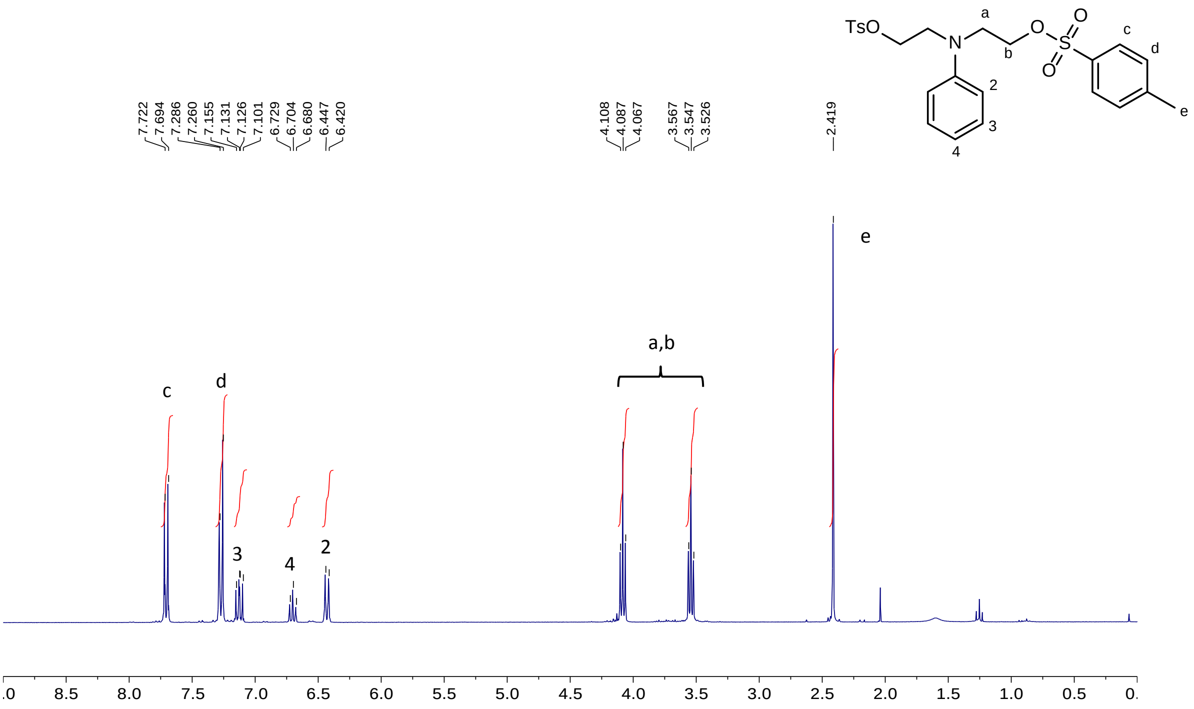

Figure S1. ^{1}H NMR spectra (300 MHz, $CDCl_3$) of compound **3**.

Compound 5.

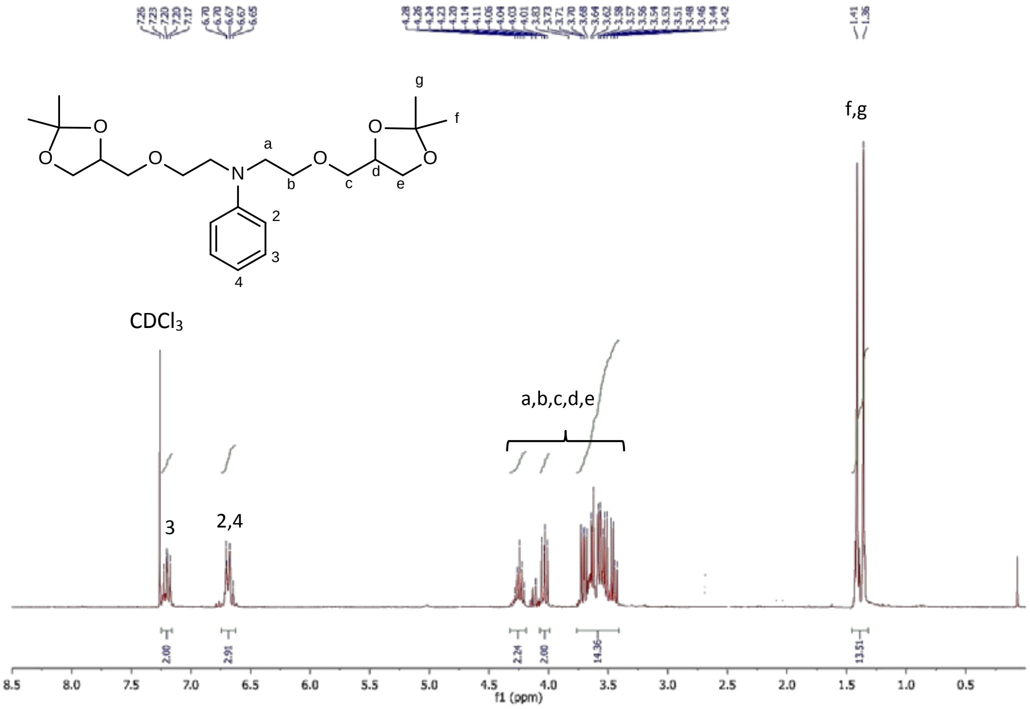

Figure S2. ^{1}H NMR spectra (300 MHz, $CDCl_3$) of compound **5**.

Compound 7.

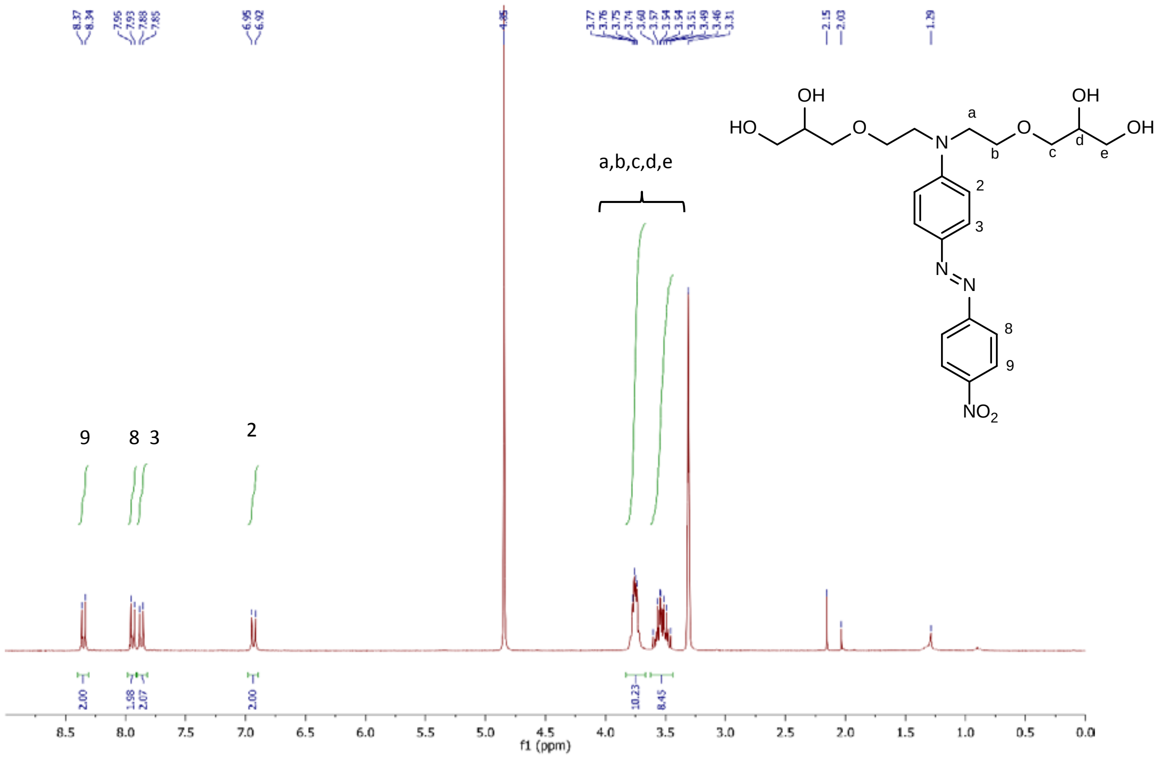

Figure S3a. ^{1}H NMR spectra (500 MHz, methanol-d_4) of compound **7**.

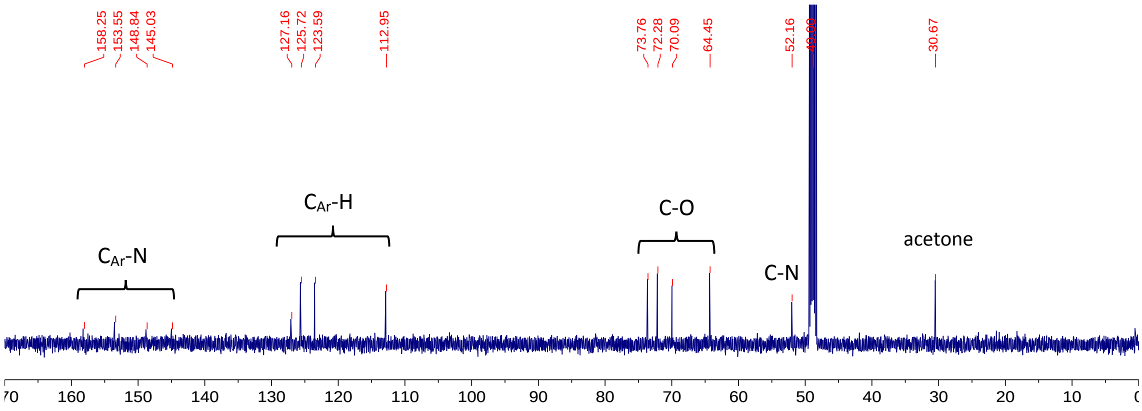

Figure S3b. ^{13}C NMR spectra (125.7 MHz, methanol-d_4) of compound **7**.

Compound 8.

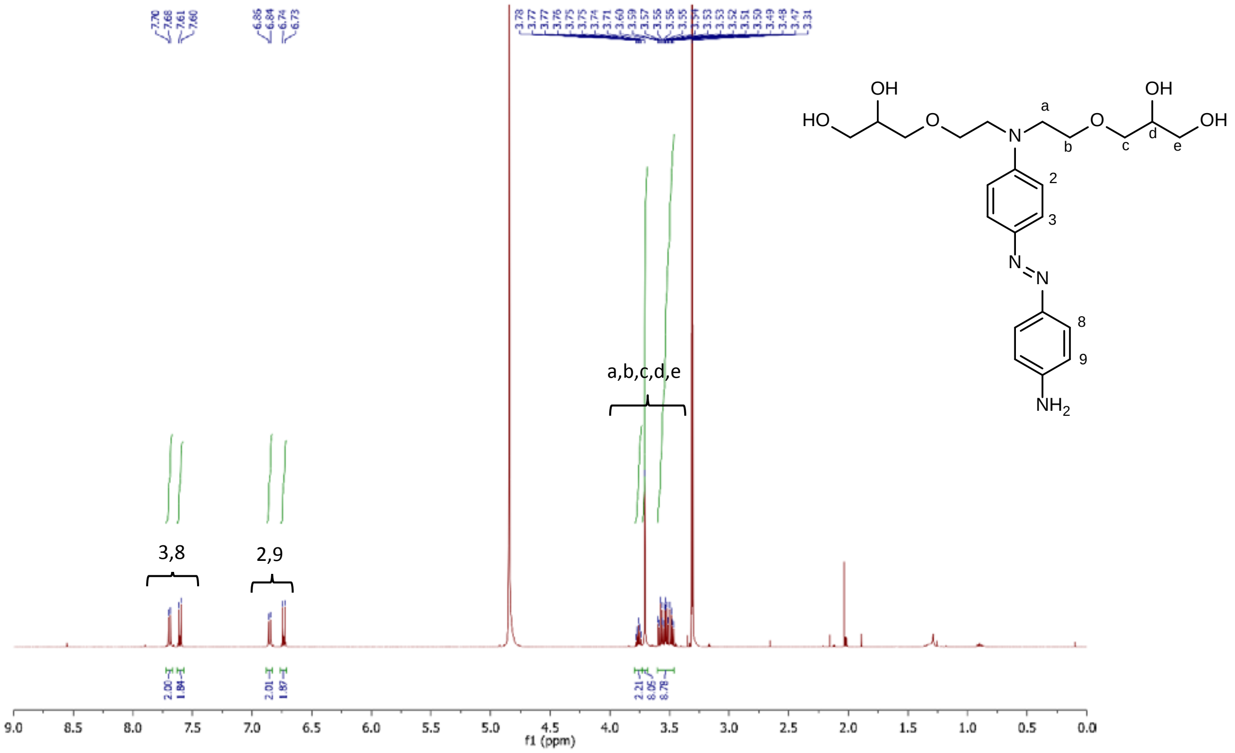

Figure S4. ^{1}H NMR spectra (500 MHz, methanol-d_4) of compound **8**.

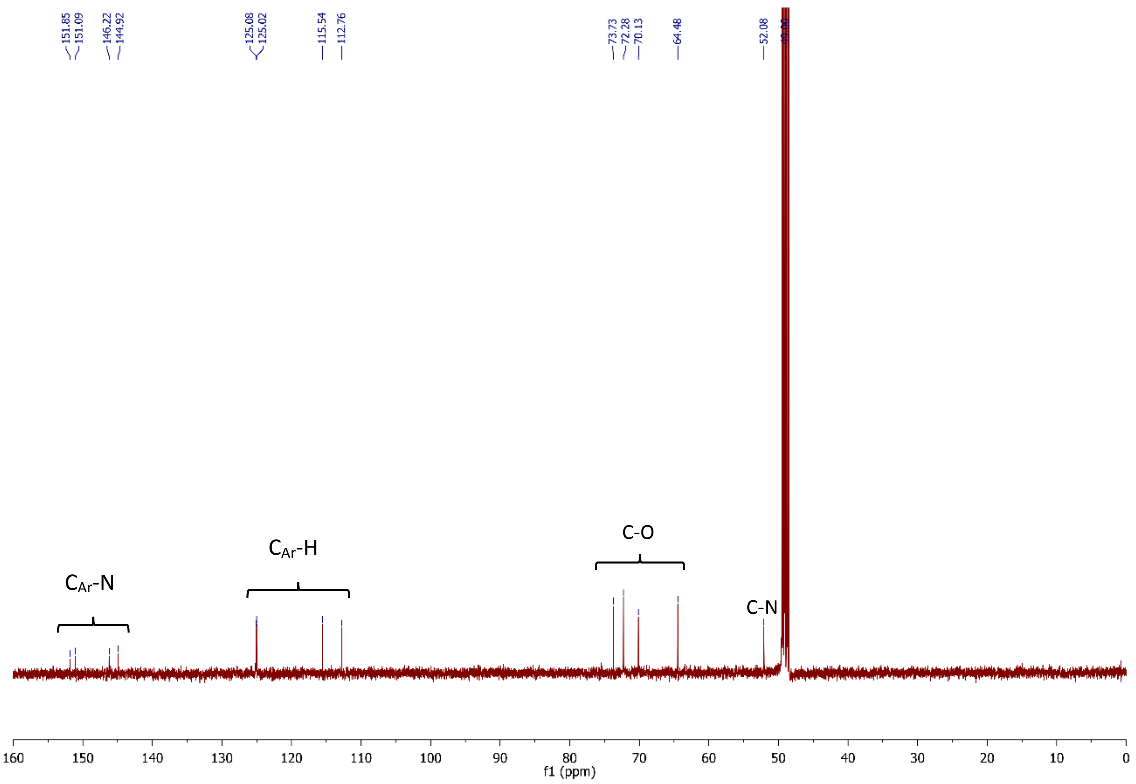

Figure S5. ^{13}C NMR spectra (125 MHz, methanol-d_4) of compound **8**.

Molecular gate 1.

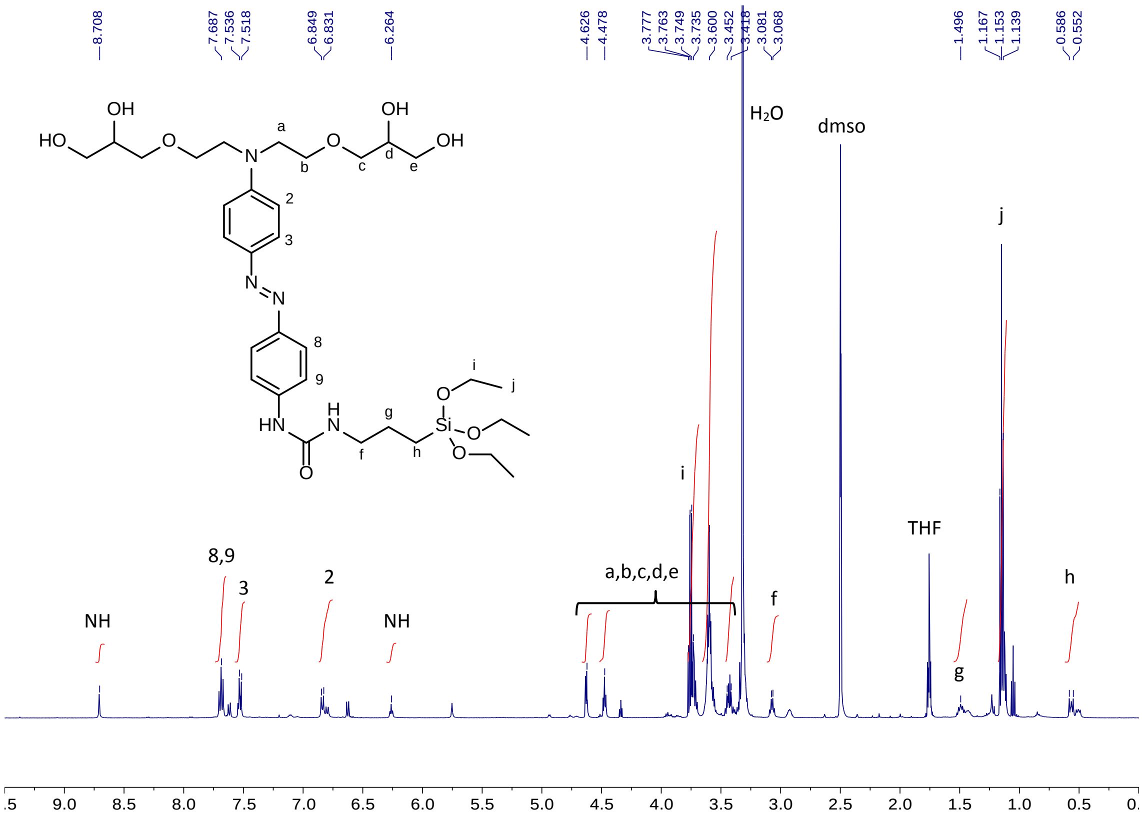

Figure S6. ^{1}H NMR spectra (500 MHz, DMSO-d_6) of compound **1**.

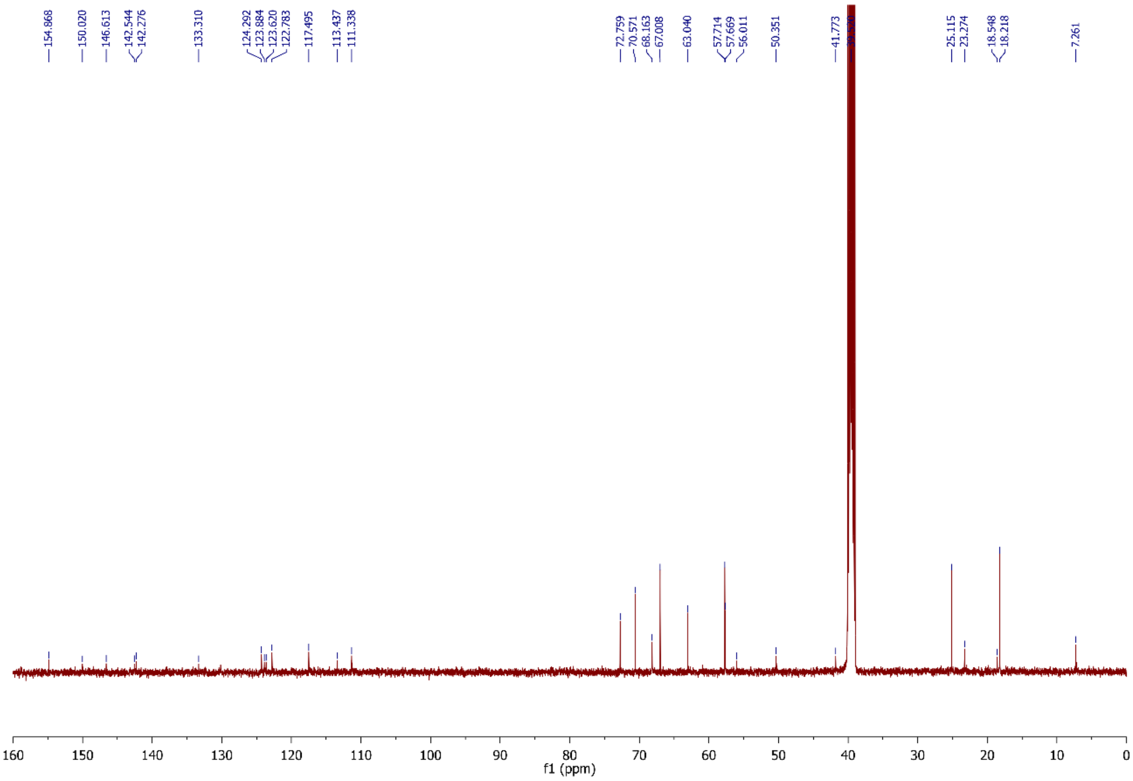

Figure S7. ^{13}C NMR spectra (125 MHz, DMSO-d_6) of compound **1**.

Laser Diffraction

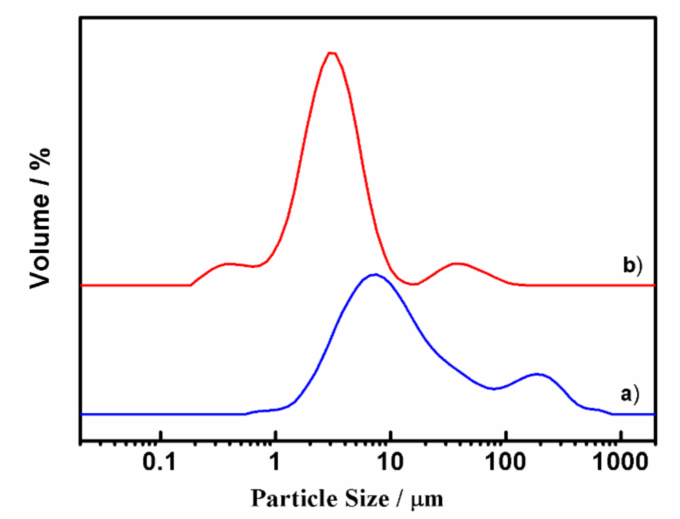

Figure S8. Laser diffraction particle size distribution of (a) **M-Bud** and b) **M-Saf** materials.

N_2 adsorption-desorption isotherms

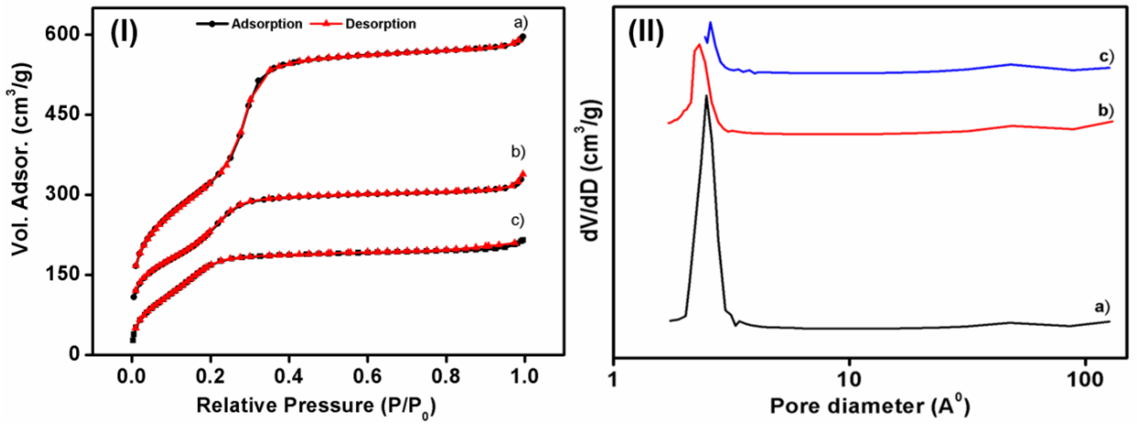

Figure S9**.** (I) N_2 adsorption-desorption isotherms and (II) BJH pore size distribution for: a) MCM-41, b) **M-Bud** and c) **M-Saf** materials.

IR spectra

a)

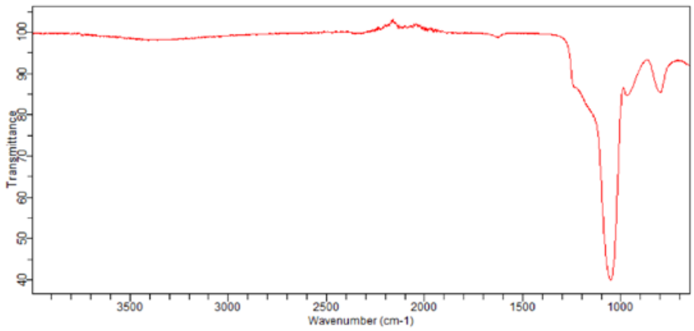

b)

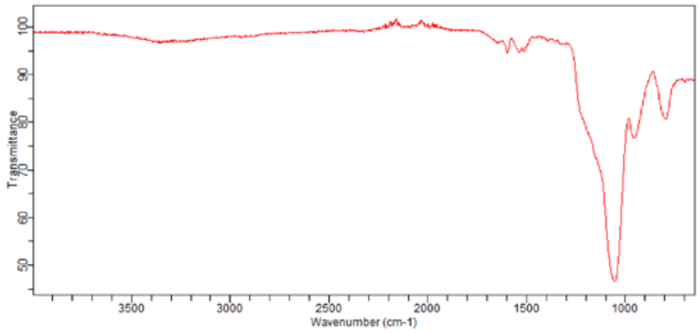

c)

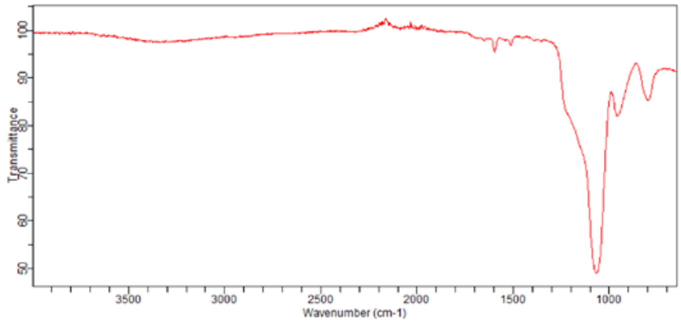

Figure S10**.** IR spectra of (a) MCM-41, (b) **M-Saf** and c) **M-Bud** materials. The bands at ca 1600 cm^{-1} in b) and c) correspond to the urea moieties

^{1}H-NMR spectrum of the reductive cleavage of compound 8

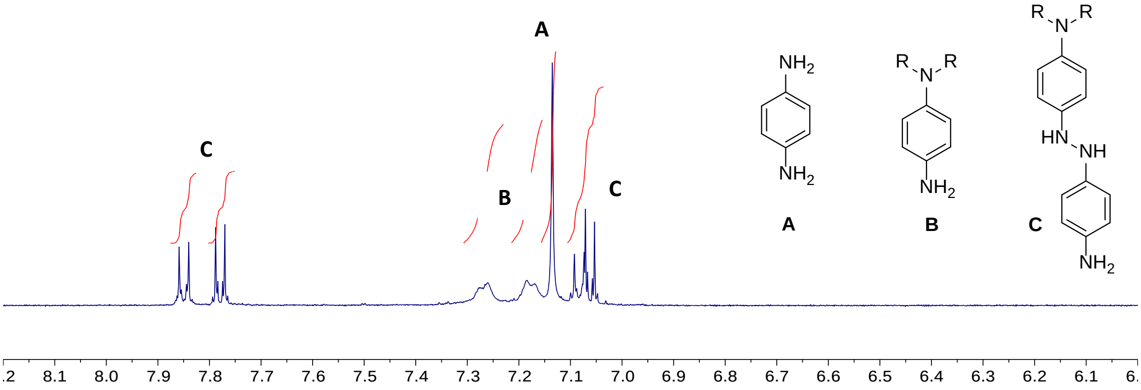

Figure S11. ^{1}H-NMR spectrum of the reductive cleavage of compound 8 after treatment with $Na_2S_2O_4$ for 20 min (D_2O, 300 MHz). No starting material was observed after 2 min reaction.

Thermogravimetric analysis and elemental analysis

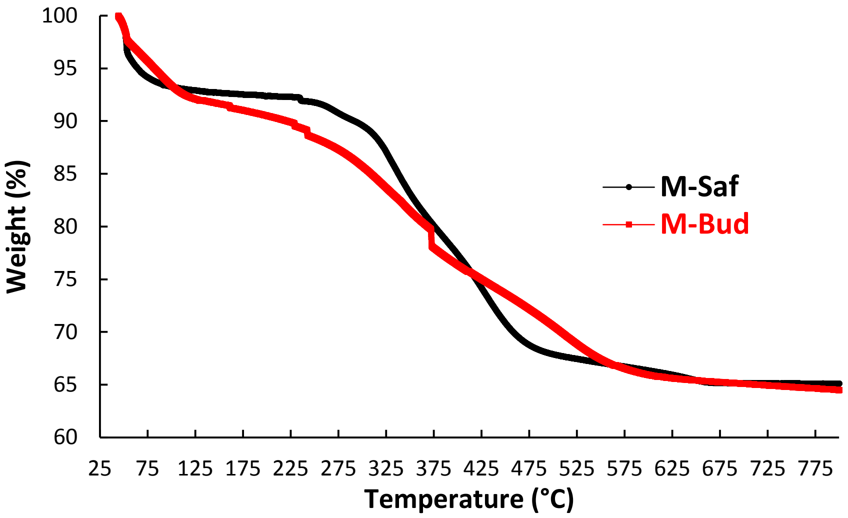

Figure S12. Thermogravimetric analysis for **M-Saf** (blue curve) and **M-Bud** (red curve), indicating ca 7-8 % H_2O and solvents, 26 % organic matter, 66-67 % SiO_2.

Table 1. Elementary analysis for selected materials.

Sample Name	Nitrogen	Carbon	Hydrogen	Sulphur
**M-Saf**	2.84	16.34	3.68	
	2.82	15.86	2.95	

Sample Name	Nitrogen	Carbon	Hydrogen	Sulphur
**M-Bud**	2.19	16.61	2.71	
	2.18	16.67	2.82	
	2.31	16.47	2.34	

HPLC conditions and procedures

The calibration method of HPLC was prepared as a previously reported.[1] The mobile phase consisted of methanol: water (80:20 v/v). The flow rate was 1 mL/min. The wavelength of detection was 244 nm (λ_{max} for BUD). The injection volume was 20 µL.

Preparation of stock and standard solutions.

Methanol: water (50:50 v/v) was used as a solvent for preparation of both stock as well as standard solutions. The stock solution of budesonide was prepared by dissolving 10.0 mg of drug in 100 mL solvent, creating a 100 µg/mL solution of budesonide. This solution was diluted with solvent a needed to prepare different standard solutions (100, 50, 25, 10, 5 and 1 µg/mL). Standard solutions each in three replicates were injected into the system. The method of linear regression was used for data evaluation. Peak area ratios of standard budesonide were plotted against theoretical concentrations of standards solutions Figure S12. Linearity was expressed as a correlation coefficient.

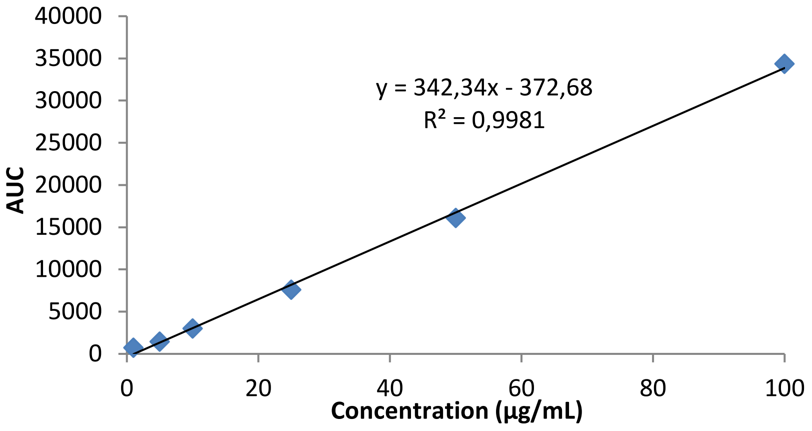

Figure S13. Standard curve of budesonide showing linearity.

Drug release studies.

In a typical experiment, 5 mg of **M-Bud** was suspended in water (10 mL), sodium dithionite (3 mg) was added and aliquots were separated after a 5 minutes and 4 hours, centrifuged. A known maximum drug release was then injected to HPLC systems. The chromatogram show a single peak at t_R = 4.20 min. and no indication of degradation of budesonide at t_R = 2.0 min. The peak area of budesonide (t_R = 4.20 min) was concentration. Drug release at 5 minutes was 24 µg/mg material and at 4 hours was 31.2 µg/mg material.

In vitro dye release studies from M-Saf at different pH values.

Dye delivery from **M-Saf** was followed by UV-vis spectroscopy monitoring the safranin O band centered at 520 nm (See Figure S14). These studies were carried out using three different buffers, to simulate the GIT, (hydrochloric acid pH 1.2, sodium acetate pH 4.5 and sodium phosphate pH 6.8) that were prepared according to the Ph Eur.

In a typical experiment, **M-Saf** (5 mg) was suspended in water (17 mL) at selected pH (6.8, 4.5 and 1.2) in the absence or in the presence of sodium dithionite (3 mg). Aliquots were taken at scheduled times, filtered off in order to eliminate the suspended microparticles and the absorbance of the safranin O released from **M-Saf** was determined. Then the filtered solution and the microparticles were returned to the initial suspension until the next measurement.

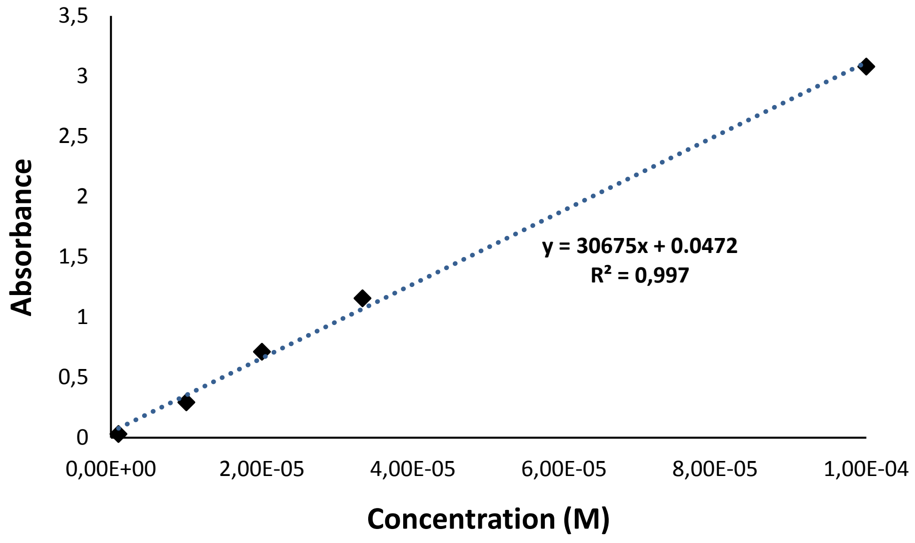

Figure S14. Plot of the absorbance at λ_{max}= 520 nm *vs* concentration for solutions of safranin O in water.

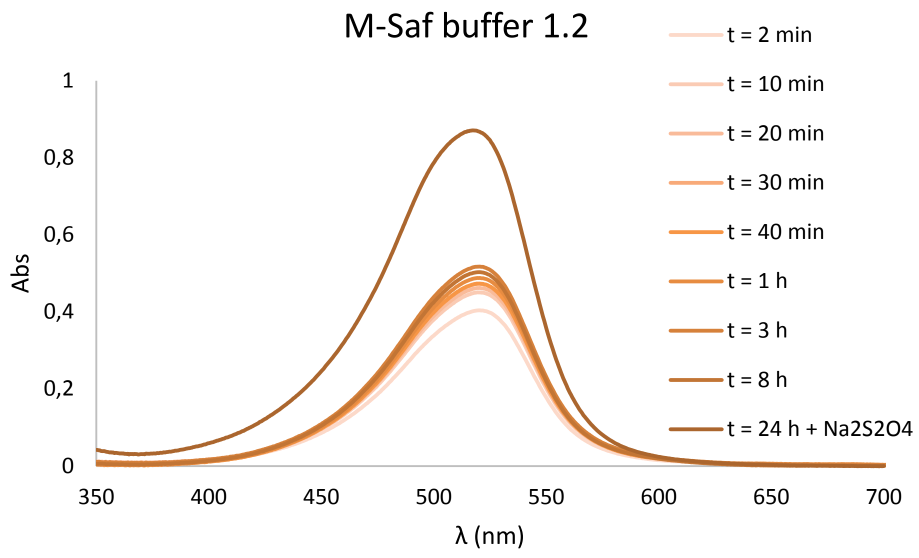

Figure S15. UV-Vis spectra of M-Saf in buffer hydrochloric acid pH 1.2.

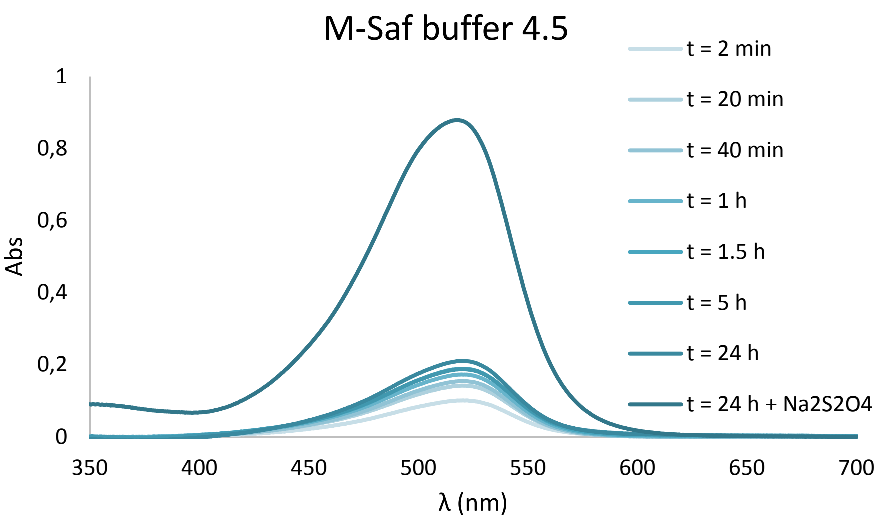

Figure S16. UV-Vis spectra of M-Saf in buffer sodium acetate pH 4.5.

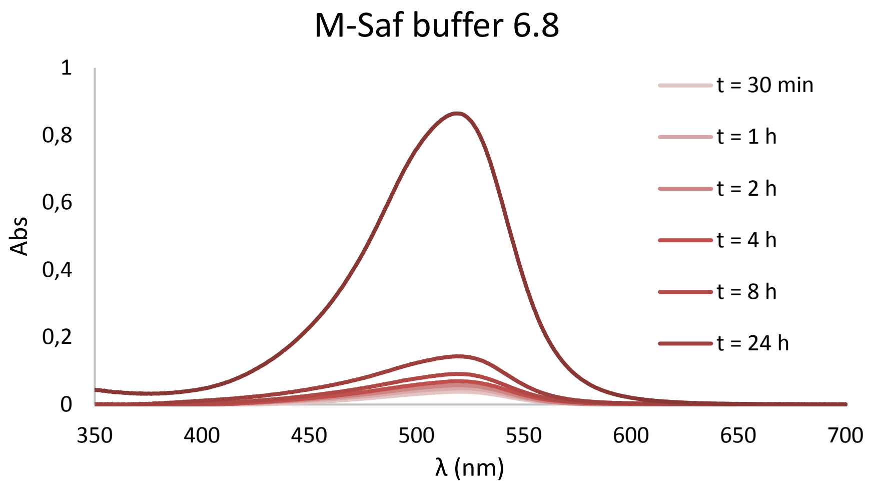

Figure S17. UV-Vis spectra of M-Saf in buffer sodium phosphate pH 6.8.

Table 2. Released cargo at different pH values and times in the absence of sodium dithionite and the same solution after the addition of sodium dithionite

**Without $Na_2S_2O_4$**								
Time	Abs	µg/mg at pH 1.2	Time	Abs	µg/mg at pH 4.5	Time	Abs	µg/mg at pH 6.8
0	0	**0**	0	0	**0**	0	0	**0**
2min	0.4041	**13.9**	2min	0.1007	**2.1**	30min	0.0386	**0**
10min	0.4506	**15.7**	20min	0.1417	**3.7**	1h	0.0479	**0.4**
20min	0.4623	**16.1**	40min	0.154	**4.2**	2h	0.0573	**0.9**
30min	0.4729	**16.6**	1h	0.1725	**4.9**	4h	0.0693	**1.7**
40min	0.4735	**16.6**	1.5h	0.1881	**5.5**	8h	0.0908	**3.7**
1h	0.4882	**17.1**	5h	0.1871	**5.4**	24h	0.1433	**3.8**
8h	0.5032	**17.7**	24h	0.2099	**6.3**			
24h	0.5033	**17.8**						

With Na_2S_2O4								
Time	Abs	µg/mg at pH 1.2	Time	Abs	µg/mg at pH 4.5	Time	Abs	µg/mg at pH 6.8
24h	1.019	**38**	24h	0.8794	**31**	24h	0.8645	**32**

Drug release studies by UV-vis.

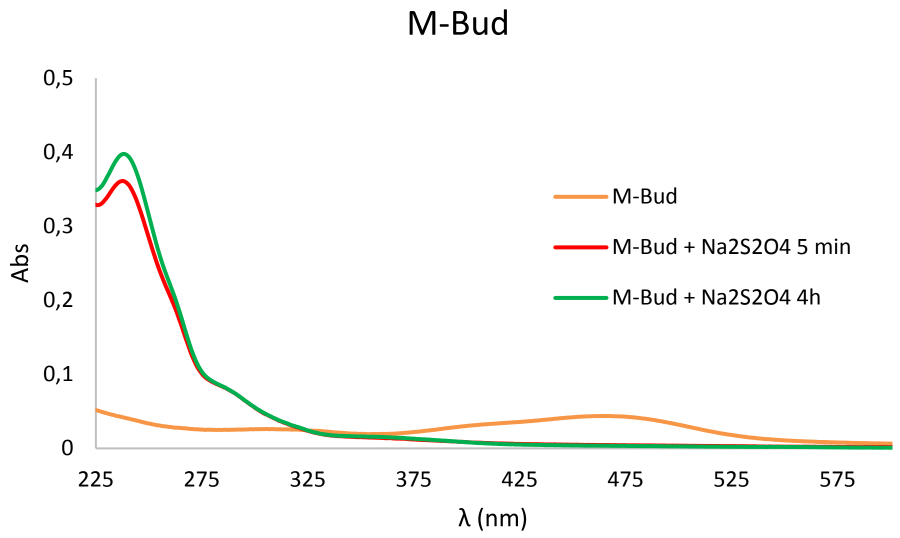

Figure S18. UV-Vis spectra of M-Bud in water (orange line) and after addition of $Na_2S_2O_4$ at 5 min (red line) and after 4 hours (green line).

***In vivo* release studies.**

The studies reported here adhere to the Principles of Laboratory Animal Care and were approved by the institutional ethics committee of the Conselleria de Agricultura, Medio Ambiente, Cambio Climático y Desarrollo Rural (Generalitat Valenciana), according to 2016/VSC/PEA/00158.

In a typical experiment, 10 mg of **M-Saf** was suspended in water (200 µL). The mixture was administrated to the mouse by oral gavage by using a cannula provided with a rounded tip. The mouse, with free access to water and food, was observed during 4 hours showing a normal behaviour. The mouse was euthanized with an overdose of anesthesia (dolethal®) and then the abdomen was opened and the digestive system was removed. In one of then, the digestive system was dissected to locate the microparticles through it. The **M-Saf** was observed to pass freely without causing any ulcer or absorption in the other tissues. After this time, an appreciable amount of closed material was present in the stomach. In addition, the microparticles located along the small intestine remained also closed. The material isolated in each section, was evaluated using sodium dithionite and the safranin O delivered was followed by fluorescence spectrometry (Figure 5 and S19). In addition, another mouse was studied during 24 h after oral administration of **M-Saf**. After 24 h the mouse was euthanized in similar

conditions and the digestive system was dissected to locate the microparticles through it. After this time, no appreciable amounts of closed material were present in the stomach, intestine or colon, and the corresponding release studies using $Na_2S_2O_4$ did not show the presence of safranin O in the extracted solutions. In addition, the stool was also evaluated. The stool was homogenized and suspended in water (5 mL) during 24 h at room temperature. The mixture was centrifuged and the supernatant was separated. The solid was suspended again in water (5 mL) and safranin O release was evaluated using sodium dithionite. After this time, safranin O was not observed (Figure S20).

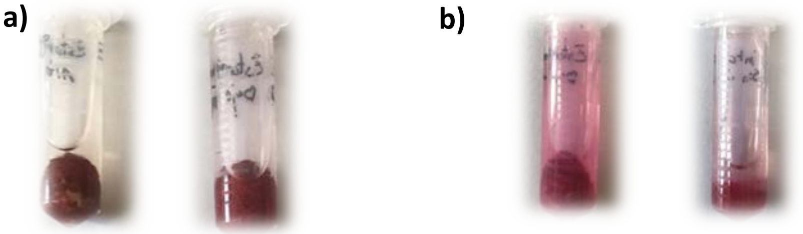

Stomac h | Intestin e | Stomach + $Na_2S_2O_4$ | Intestine + $Na_2S_2O_4$

Figure S19. The **M-Saf** located along the stomach and small intestine after 4 h in the absence (a) and in the presence (b), of $Na_2S_2O_4$

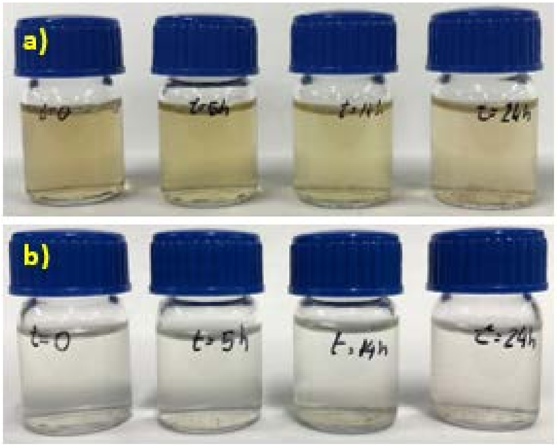

Figure S20. The stool supernatant water extraction after 24 h in the absence (a) and in presence (b) of $Na_2S_2O_4$.

References

1. S. R. Naikwade, A. N. Bejaj, P. Gurav, M. M. Gatne, P. Soni, *AAPS Pharm. Sci. Tech*, **2009**, 10, 993-1012.
